# Supplementary material for: Auxin confers protection against ER stress in Caenorhabditis elegans
Source: Biol Open. 2021 Feb 3;10(2):bio057992. doi: 10.1242/bio.057992 (PMC7875485; doi:10.1242/bio.057992)
Supplement: Supplementary information [file biolopen-10-057992-s1.pdf]

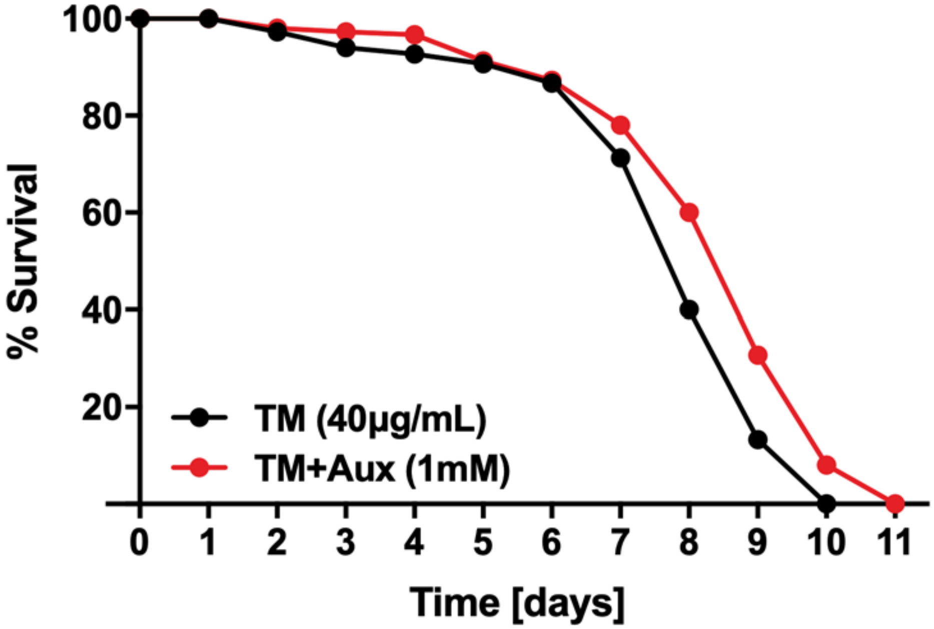

Figure S1. Auxin induces ER stress resistance in adult worms. Repeat of the experiment shown in figure 2. Survival of adult wildtype worms on plates containing tunicamycin (40 µg/mL) alone (N=153) or tunicamycin and auxin (1 mM) together (*P*<0.0001) (N=154). The P value was calculated using the log-rank (Mantel-Cox) method. Mean and maximum lifespan were 8.1 and 10, and 8.6 and 11, for tunicamycin and tunicamycin/auxin, respectively.
